# The spatial linkage mechanism: medical level, public health security, and economic climate from 19 OECD EU countries

**DOI:** 10.3389/fpubh.2023.1090436

**Published:** 2023-06-26

**Authors:** Rong Fu, Binbin Zheng, Tao Liu, Luze Xie

**Affiliations:** ^1^College of Economics, Hangzhou Dianzi University, Hangzhou, China; ^2^Department of Sociology, Hangzhou Dianzi University, Hangzhou, China

**Keywords:** COVID-19, public health, economic climate, economic growth, dynamic spatial Durbin model, OECD

## Abstract

**Introduction:**

The global spread of the COVID-19 has brought about global changes, especially in terms of economic growth. Therefore, it has become a global issue to explore the impact of public health security on the economy.

**Methods:**

Employing a dynamic spatial Durbin model, this study analyzes the spatial linkage mechanism of medical level, public health security, and economic climate in 19 countries as well as investigates the relationship between economic climate and COVID-19 by the panel data of 19 OECD European Union countries from March 2020 to September 2022.

**Results:**

Results show that an improvement in the medical level can reduce the negative impact of public health security on the economy. Specifically, there is a significant spatial spillover effect. The degree of economic prosperity hurts the reproduction rate of COVID-19.

**Discussion:**

Policymakers should consider both the severity of the public health security issues and the economic level when developing prevention and control policies. Given this, corresponding suggestions provide theoretical support for formulating policies to reduce the economic impact of public health security issues.

## 1. Introduction

At the beginning of 2020, the COVID-19 pandemic affected national economic growth and individuals’ basic life necessities. It has been the largest global public health crisis since the influenza pandemic in 1918 (1) and is still prevalent worldwide. Under the COVID-19 pandemic, global change is inevitable.

Thus, it has become urgent to analyze and interpret the impact of the COVID-19 pandemic as well as what measures should be implemented to handle similar crises. Similar to the COVID-19 pandemic, the SARS outbreak in 2003 had a profound impact on the Asian tourism industry and affected the economic growth of Asia and the world. At the same time, it showed that the ban and measures adopted can effectively prevent the spread of the virus, but they may exacerbate panic (2). From the data available now, the impact of SARS is much smaller than our estimate of its current occurrence (3). The features that differentiate COVID-19 from recent encounters are its wide geographical spread in terms of contagion and its high mortality rate (4). Compared to the impact of the SARS outbreak, the economic impact and spillover effects on the European Union (EU) increased significantly when the COVID-19 pandemic occurred. China’s economic growth was affected at the initial stage of COVID-19, but it soon recovered. Thanks to the increase in China’s influence, the country was less affected by the negative spillover effects of other countries during the pandemic (5). Accordingly, we can maintain a wait-and-see attitude for the current prediction of losses caused by the COVID-19 pandemic.

### 1.1. COVID-19 pandemic and economic growth: a historical overview

The most obvious harm of global change caused by the COVID-19 pandemic is the increased medical burden. In Italy, for example, the number of deaths due to COVID-19 in the first quarter of 2020 reached 18,000, resulting in a loss of economy and productivity (6). The levels of anxiety, depression, and stress of healthcare employees were also significantly affected (7, 8). In the São Paulo intensive care ward, Brazil, the rate of hand hygiene during the COVID-19 pandemic decreased significantly compared to that before the pandemic. This was because the increased medical burden caused by the pandemic and other diseases had not resulted in increased hand hygiene compliance (9). Moreover, the high cost of COVID-19 treatment has exacerbated the existing burden on developing countries. At the same time, economic growth also affects the regional virus transmission rate. Especially in areas with unfavorable economic development, the transmission rate of COVID-19 cannot be well controlled. This situation has formed a vicious circle of the poorer the more serious, the more serious the poorer (10). Developed countries have more medical experience and higher medical standards, which has enabled them to the second and third waves of the handle pandemic better than developing countries (11). On this basis, the national government of developing countries can solve the problems through reasonable measures to reduce the cost of treatment and implement tiered charges for both rich and poor areas (12). The indirect impact of the COVID-19 pandemic is also huge, especially its negative impact on economic growth. Under the conditions of the COVID-19 outbreak, the medical capacities of hospitals were lacking, which inevitably led to economic losses. The cost of adopting different means of prevention and control of COVID-19 varies, and isolation may be the best way to deal with it (13).

In addition to social isolation, the national government should introduce policies to control the spread of COVID-19. Under the COVID-19 transmission model, every country will be affected by economic conditions and government intervention measures. Good economic conditions will exacerbate the spread of COVID-19, while appropriate government intervention measures will greatly reduce its spread (14). Specifically, the COVID-19 pandemic has reduced the activities of the service and manufacturing industries, resulting in an increase in the number of unemployed people. To reduce the negative impact of the pandemic and intervention measures on the economy, the government should take financial, monetary, and other economic measures to expedite economic recovery (15, 16). At a time when rapid antigen diagnostic tests (RTDs) are widely used in COVID-19 detection, the government can turn the COVID-19 pandemic into a controllable infection through rapid testing (17). Standing at the crossroads of this choice, governments should learn a lesson. It is important to revive the economy, but once everyone is dead, then no one contributes to the economy (18). In emergency response, it is usually better for the government to overreact and then scale down when necessary, rather than to react too late (19). Therefore, the government must grasp the intensity of intervention, both development and pandemic prevention and control. Health spending can affect GDP to some extent, and its impact is not entirely linear; increased health spending in a country increases human capital, either directly or indirectly, leading to higher productivity and an increase in GDP (20, 21). Healthcare levels and economic development go hand in hand. For example, Bangladesh’s economic growth is hindered by underdeveloped medical care (22). Therefore, the government should increase medical funding to mitigate the impact of the COVID-19 pandemic on residents’ lives and economic growth (23). COVID-19 affects not only current economic growth but also the future expectations of investors and consumers. Taking the United States as an example, the study finds that the health crisis and economic downturn will have a negative impact on investors. At the same time, the health crisis in other countries will also have a negative spillover effect on investor expectations (24). The economic recession brought about by the COVID-19 pandemic will inevitably affect the unemployment rate, which will in turn reduce national tax revenue and increase government spending. Therefore, the federal government must avoid major deficits and harmful cuts, and improve the healthcare safety net by increasing Medicaid (25).

### 1.2. Strategic measures under COVID-19

The relationship between economic growth and the COVID-19 pandemic is complex. In the long run, the harm caused by the pandemic to the country and society is continuous, but the positive environmental impact is only temporary. The outbreak of COVID-19 has slowed or even stalled the global economy, reduced carbon emissions, and improved air quality in many cities around the world. However, when the pandemic subsides, carbon and pollutant emissions will return to the same levels as before, and the positive environmental impact of the pandemic will be lost (26). The mutation and invasion of COVID-19 strains require government departments to develop more powerful strategies to overcome the threat caused by COVID-19 (27). In this regard, Akighir et al. (28) estimated the macroeconomic development level of Nigeria after adopting the economically sustainable development plan, indicating that the sustainable economic development policy of the government has a positive impact on national economic growth, employment, inflation, and so forth. Dorn et al. (29) studied a balanced strategy that can meet the co-benefits of health protection and the economy, and also reduce economic losses without compromising medical goals. Contrary to the economic pain caused by the COVID-19 pandemic to the general public and the non-investment class, the market has brought immeasurable rewards to those at the top (30). For example, the pharmaceutical industry benefitted greatly from the shortage of drugs in the early stage of the pandemic (31). The development of health tourism has also helped the economic growth of countries such as Turkey. The national government can alleviate the economic losses caused by COVID-19 by supporting health tourism (32).

### 1.3. Aims and contributions of the study

As the most influential public health security issue at present, COVID-19 has had an impact on all aspects of the world. Especially in the economic aspect, the pandemic has brought a huge blow to the economy. With globalization enhancing international exchanges, the spillover effect of the pandemic cannot be ignored. Many studies have used different models to study the relationship between COVID-19 and the economy (33–35), especially the direct impact of the pandemic on the economy. Different medical levels between countries lead to differences in the way and effectiveness of pandemic prevention in each country, and the medical level becomes an influential factor during a pandemic. The present study combines the economic level matrix and the geographical distance matrix to build a dynamic spatial Durbin model (36). We are more concerned with the indirect impact of the pandemic on neighboring countries. The dynamic spatial Durbin model can well integrate geographical distance into the model, expand the impact of the COVID-19 pandemic from one country to neighboring countries, and more comprehensively describe the spread and harm of the pandemic in the context of globalization. Expanding from COVID-19 to general public health security issues informs future public health security issues. Based on this, the study proposed two main hypotheses.

*Hypothesis 1*: There is a spatial spillover effect of the medical level on public health safety issues and economic climate.

*Hypothesis 2*: Increased public health safety issues can inhibit current economic growth; however, current economic growth cannot alleviate public health security issues.

This paper only considers the extent to which the medical level and the strictness of policy response affect the public health safety issues and the economy. Other variables do not have a significant impact on the model. Therefore, the study assumes that other variables such as demographic structure, industrial structure, and psychological factors are not significantly different across countries. Moreover, differences in topography and landscape across countries do not affect the construction of the geographical distance matrix.

Compared with the existing literature, this paper possibly makes the following contributions: first, adopting the COVID-19 disability-adjusted life year (DALY) as a public health safety (PHS) indicator and using it to analyze the impact of public health safety issues on the economic climate; and second, adding control variables to the Durbin model (strictness of policy response), which reduces the impact of different national pandemic prevention policies on the results.

## 2. Materials and methods

### 2.1. Variable selection and data interpretation

This study selects data from the official statistics of the OECD and the data provided by Martin College of Oxford University and the Global Clinical Development Lead. In particular, it uses the Comprehensive Leading Indicator (CLI) to measure the degree of future economic prosperity; the quadratic interpolation to convert quarterly GDP into monthly GDP, which is then taken as the economic climate level of each country; and the COVID-19 DALY as a public health safety (PHS) indicator. Three control variables are selected: medical level (ML), which is the number of hospital beds per 1,000 people in each country, is selected as a measure of the medical level of each country during the COVID-19 pandemic; policy response strictness (PRS) is the monthly average government response strictness index; and reproduction rate (RR) is the country’s monthly virus reproduction rate.

### 2.2. Public health safety calculation

Most infectious diseases that broke out in recent years have the characteristics of widespread contagion and profound impact. Many human infectious diseases have evolutionary patterns, such as AIDS, malaria, and hepatitis B. Their initial appearance led to a pandemic, with periodic outbreaks experienced in the process of human society, eventually forming endemic diseases and likely to erupt in the future (37). Especially in the process of globalization, geographical restrictions have weakened, and infectious diseases such as tuberculosis have moved from high-prevalence areas to low-prevalence areas with the deepening of international exchanges, thus affecting global public health security (38). COVID-19 has also caused a severe blow to tourism, hotel, education, and other industries (39–41). In addition, the losses caused by the pandemic do not only exist in the present. For instance, many patients have developed long-term physical and even psychological problems due to the disease. Indeed, the impact of the disease is prolonged. For a long time, the incidence of infectious diseases alone could not accurately measure the regional PHS indicators. Compared to disease incidence and mortality, disease burden more comprehensively reflects the regional disease severity and regional public health security level. Disease burden refers to the economic, life, and quality of life loss of patients after the occurrence of the disease (42). Moreover, when the Harvard T.H. Chan School of Public Health, the World Bank, and the World Health Organization (WHO) cooperated to assess the global disease burden in 1993, a new indicator of DALY was introduced in assessing the global disease burden (43). Since then, the DALY measurement has become a common disease burden measurement method. In view of this, the present research uses DALY to measure disease burden and constitute a PHS index. The construction process is as follows (44):


(1) DALY=YLD+YLL

In Formula (1), *YLD* represents the number of years of health lost due to disability, and *YLL* represents the number of years of life lost due to death.


(2) $$YLD=\frac{I\times DW\times L\left(1-e^{-rL_{D}}\right)}{r}$$

(3) $$YLL=\frac{N}{r}\left(1-e^{-rL}\right)$$

In Formula (2), *I* represent the number of patients infected by COVID-19 (this study adopts the number of cases per 100,000 people), and *DW* represents the weight of the disease. According to the research on the weight of the disease proposed by Saloman et al. (45), the *DW* of the COVID-19 pandemic is equal to 0.133; $L_{D}$ is the disease duration, usually two weeks, or 0.0038 years; and *r* is the discount rate, usually 0.03. In Formula (3), *N* represents the number of deaths due to COVID-19 (this study adopts the number of deaths per 100,000 people), and *L* represents the life expectancy. The final estimated DALY included panel data of 31 months for 19 countries.

### 2.3. Spatial correlation test

The basic measure of spatial autocorrelation analysis is Moran’s I, which is derived from the Pearson correlation coefficient in statistics and can reveal the laws of geographic space (46). In this research, Moran’s I is used to test the spatial correlation of the indicator of the economic climate. The statistical calculation process of the global Moran’s I to measure the spatial correlation is as follows:


(4) $$I=\frac{n\sum_{i=1}^{n}\sum_{j=1}^{n}W_{i,j}\left(Y_{i}-\bar{Y}\right)\left(Y_{J}-\bar{Y}\right)}{S_{0}\sum_{i=1}^{n}{\left(Y_{i}-\bar{Y}\right)}^{2}}$$

In Formula (4), $Y_{i}$ is the OECD CLI of each country, $W_{i,j}$ is the economic geospatial weight between countries i and j, n is the total number of countries, and $S_{0}$ is the aggregation of spatial weights:


(5) $$S_{0}=\sum_{i=1}^{n}\sum_{j=1}^{n}W_{i,j}$$

Since countries within the EU are not only connected by distance but also by mutual economic activities, an economic geospatial nested matrix is constructed (47). The construction process is as follows:


(6) $$w_{i,j}^{1}=\left\{ \begin{array}{l} \;0\;\;if\;i=j,\\ \frac{1}{d_{i,j}^{2}}if\;i\neq j, \end{array}\right.$$

(7) $$w_{i,j}=w_{i,j}^{1}diag\left(y_{1},y_{2},\cdots ,y_{n}\right)$$

where $y_{i}$ refers to the GDP *per capita* of each country from 2020 to 2021 (calculated by purchasing power parity), $d_{i,j}$ refers to the distance between national capitals (the unit is kilometers), and $w_{i,j}$ is standardized on the basis of Formula (7):


(8) $$W_{i,j}=\frac{w_{i,j}}{\sum_{j=1}^{n}w_{i,j}}$$

By Formula (8), the $w_{i,j}$ matrix is made dimensionless to make it reflect the spatial correlation structure more clearly, which is convenient for the subsequent drawing of the local Moran exponent map and the establishment of the spatial Durbin model.

**Figure 1:**
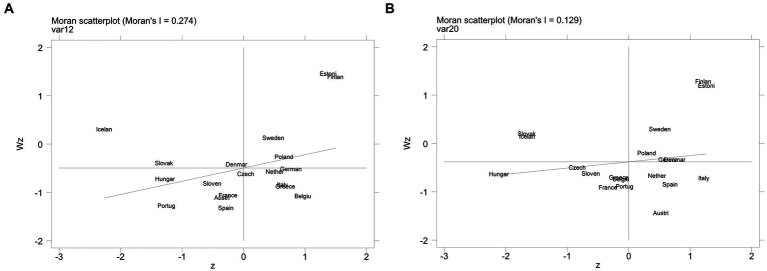
Lisa scatterplot of the AnselinLocal Moran’s I. **(A)** Lisa scatterplot in February 2021; **(B)** Lisa scatterplot in November 2021.

From August 2020 to February 2022, Moran’s I is positive and significant (Table 1), indicating that the CLI of European countries has a significant positive correlation in the spatial distribution, that is, there is a clustering trend in the economic growth of neighboring countries, and the spatial clustering effect really exists. This paper then draw a LISA scatterplot of the Anselin Local Moran’s I (Figure 1). The cross-sectional data in February 2021 and November 2021 are analyzed, and it is found that there were high-high aggregation and low-low aggregation between countries, with a strong spatial clustering effect.

**Table 1:** Global Moran’s I from March 2020 to September 2022.

Month	Moran’s I	Month	Moran’s I
2020/03	−0.024	2021/07	0.658^*^
2020/04	−1.214^**^	2021/08	0.675^*^
2020/05	−0.996^**^	2021/09	0.735^*^
2020/06	−0.812^*^	2021/10	0.746^*^
2020/07	−0.034	2021/11	0.675^*^
2020/08	0.111	2021/12	0.525
2020/09	0.283	2022/01	0.306
2020/10	0.555	2022/02	0.043
2020/11	0.767^*^	2022/03	−0.200
2020/12	0.941^**^	2022/04	−0.368
2021/01	1.156^***^	2022/05	−0.459
2021/02	1.211^***^	2022/06	−0.476
2021/03	0.998^**^	2022/07	−0.407
2021/04	0.905^**^	2022/08	−0.328
2021/05	0.788^**^	2022/09	−0.252
2021/06	0.670^*^		

*10% significance level, **5% significance level, ***1% significance level.

### 2.4. Constructing the dynamic spatial Durbin model based on the moderating effect theory

If the relationship between variables X and Y is represented by a function with variab+le M, then M is the moderating variable (48). Compared to the interaction effect, the independent variable and moderating variable in the moderating effect are asymmetric and cannot be interchanged. The current study adopts the most commonly used adjustment model proposed by Wen et al. (49) to analyze the adjustment effect of medical level on public health security issues. The specific test equation is as follows:


(9) Y=aX+bM+cXM+e

Since there is a time lag in the degree of economic prosperity, the time lag term of the degree of economic prosperity is introduced into the standard static spatial Durbin model. The exogenous variable PRS is introduced as a control variable, and a dynamic spatial Durbin model is established based on the adjustment effect (50):


(10) $$\begin{array}{l} Y_{i,t}=\beta_{0}+\beta_{1}Y_{i,t-1}+\delta_{1}\sum_{j=1}^{n}W_{i,j}Y_{j,t}+\beta_{2}PHS_{i,t}+\delta_{2}\sum_{j=1}^{n}W_{i,j}PHS_{j,t}\\ +\beta_{3}ML_{i,t}+\delta_{3}\sum_{j=1}^{n}W_{i,j}ML_{j,t}+\beta_{4}PHS_{i,t}\times ML_{i,t}+\delta_{4}\sum_{j=1}^{n}W_{i,j}PHS_{j,t}\times ML_{j,t}\\ +\beta_{5}PRS_{i,t}+\delta_{5}\sum_{j=1}^{n}W_{i,j}PRS_{j,t}+\beta_{5}RR_{i,t}+\delta_{5}\sum_{j=1}^{n}W_{i,j}RR_{j,t}+\mu_{i}+\varepsilon_{i,t} \end{array}$$

In Formula (10), *Y* is the explained variable future economic climate (CLI) and economic climate (GDP). The degree of PHS is the explanatory variable, ML is the moderating variable, and PRS and virus RR are the control variables.

The following model tests are based on a mixed panel data model with the interaction term removed from the model set up above. On this basis, the LM test is carried out to show the rationality of choosing the spatial Durbin model. As shown in Table 2, both the spatial lag model test and spatial error model test are significant, indicating that both models are supported and the mixed OLS model is rejected. Thus, the rationality of using the spatial Durbin model is confirmed.

**Table 2:** Test results of traditional mixed panel model.

Test	CLI	GDP
LM-lag	50.950^***^	567.365^***^
LM-error	59.799^***^	536.225^**^
Hausman	37.07^***^	34.61^***^

**5% significance level, ***1% significance level.

Then, the Hausman test is carried out on whether the model adopts the fixed-effect model or the random-effect model. The *p* value of the Hausman test is less than 0.05, which proved that the fixed-effect model should be selected. The LR test and Wald test are then carried out on the model to analyze whether the spatial Durbin model will degenerate into a spatial auto-regression model and a spatial error model, which shows that the choice of the spatial Durbin model is very reasonable. The results are shown in Table 3.

**Table 3:** Test results of the fixed effect model.

Test	CLI		GDP	
LR-test	SDM-SAR	SDM-SEM	SDM-SAR	SDM-SEM
	18.52^***^	7.97^*^	49.05^***^	107.32^***^
Wald-test	Unconstrained	Constraint	Unconstrained	Constraint
	18.99^***^	8.12^*^	49.01^***^	65.23^***^

*10% significance level, ***1% significance level.

### 2.5. Panel model of interaction mechanism

The impact of the COVID-19 pandemic on national economic growth is described above, but the impact between the two is mutual. Health infrastructure, pandemic prevention, and control policies, urban density, urban environment (51), and economic growth will all have an impact on the severity of the COVID-19 pandemic in a region. Structural changes have an important impact on the discovery of causality, for example, Xu et al. (52) found that economic activities mainly caused environmental pollution through the shock of the COVID-19 pandemic. This paper refers to Guven et al. (53) fixed-effect panel model to reset the model and further analyze the impact of economic growth level on the severity of the pandemic. After the Hausman test, it can be seen that the fixed effect model is more suitable, and so the following fixed effect model is set:


(11) $$RR_{i,t}=\beta_{0}+\beta_{1}Y_{i,t}+\beta_{2}Z_{i,t}+\mu_{i}+\varepsilon_{i,t}$$

The dependent variable is the regional COVID-19 pandemic severity (RR). $Z_{it}$ represents the control variables PHS and PRS, and $Y_{i,t}$ represents the future economic climate (CLI) and economic climate (GDP). After testing, it is known that the inflation factor of each explanatory variable is less than 10, so there is no multicollinearity.

## 3. Results

### 3.1. Comparative analysis of Spanish flu and COVID-19

As the most lethal infectious disease in human history, the Spanish flu changed human life. Like COVID-19, which is currently prevalent, hatred makes people regard a country as the culprit. Most infected people have low immunity due to illness and die of other diseases (54). Both pandemics have a severe blow to the global medical and healthcare system. In the early days of pandemics, when confronted with these two pandemics, people underestimated the infection and mortality rates of the viruses associated with them (55). Therefore, this study compares the two pandemics (56).

As shown in Table 4, the death toll of the Spanish flu was higher than that of COVID-19, due to war and low medical levels at that time. The current medical level is far higher than that in 1918, but the proportion of deaths in the total is still not low. We can see the changes in the scope of influence: the world links closely in the context of globalization. The scope of influence of COVID-19 is much larger than that of the Spanish flu. The economic losses from COVID-19 were much higher than those from the Spanish flu due to the globalization of the economy, and the spillover from the COVID-19 pandemic has dealt a severe blow to the global economy. Therefore, the spillover effect between regions needs to be considered in public security research to prepare for the next attack on public health security.

**Table 4:** Comparison between COVID-19 and Spanish flu.

	COVID-19	Spanish Flu
Influenza duration	December 2019 -	25 months
Scope of influence	Global	Less than half of the countries
Main age group of dead patients	Age more than 65 years	25–40 years old
Proportion of deaths to total population (Italy)	0.2%	5%
Economic loss (Mexico)	180 billion dollars	9 billion dollars

The number of Spanish flu deaths in Italy is based on the study of Burdekin (57).

### 3.2. Comparison of public health security among countries

**Figure 2:**
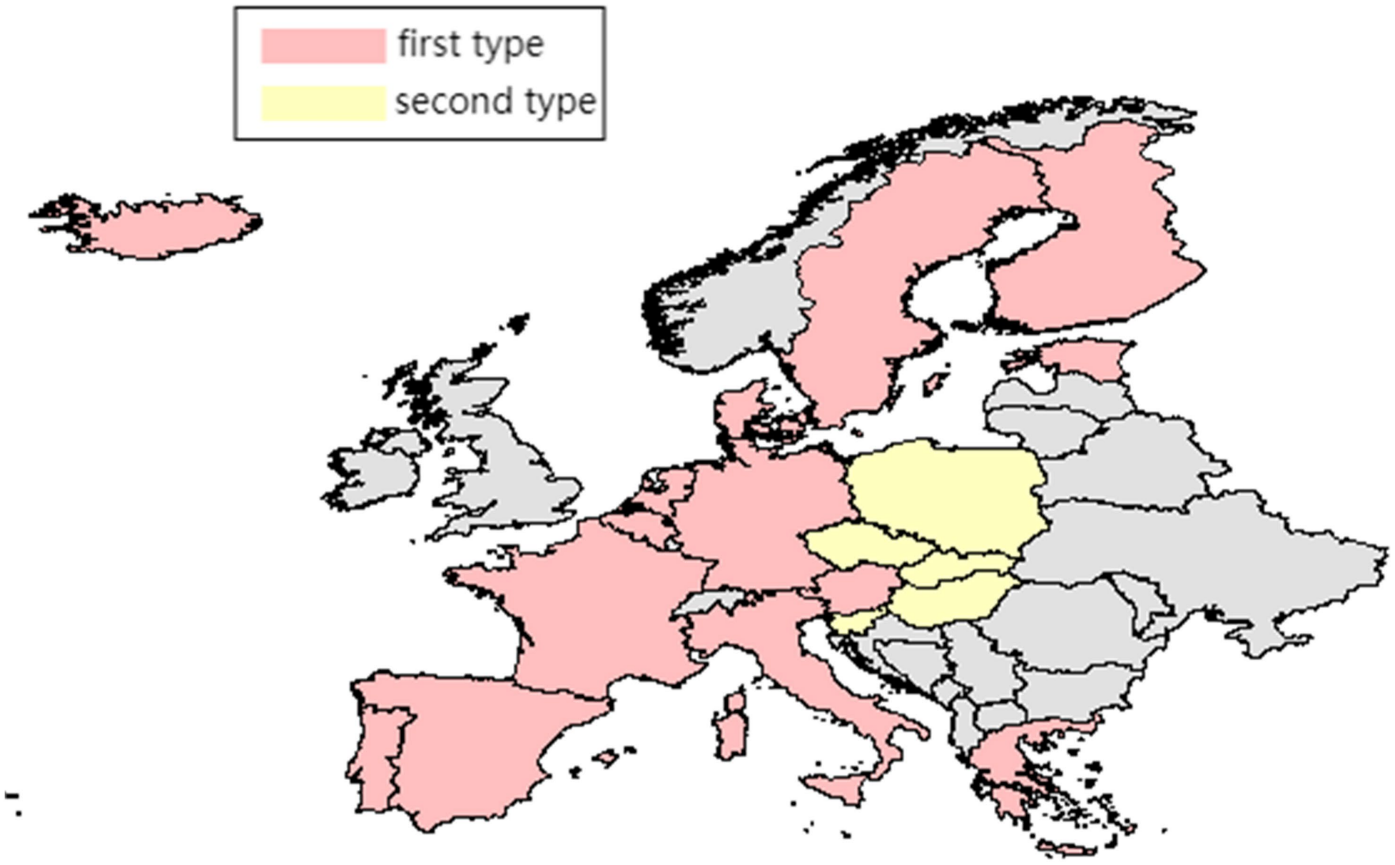
Geographical location map of two kinds of countries.

Clustering analysis was performed on the PHS indicators of each country with the time period as a variable to judge the similarity of the degree of impact of the COVID-19 pandemic among countries as well as analyze whether the similarity is related to geographical distance. These indicators are clustered using the silhouette coefficient method and K-means clustering method. Using the silhouette coefficient method, it is known that the optimal number of classifications is divided into two categories. The classification results are Austria, Belgium, Denmark, Estonia, Finland, France, Germany, Sweden, Iceland, Italy, the Netherlands, Portugal, Spain, and Greece for the first category; and the Czech Republic, Hungary, Poland, Slovakia, and Slovenia for the second category. As shown in Figure 2, the second type of countries are relatively close in geographical location and primarily concentrated in central Europe, while the first type of countries is primarily concentrated in western Europe and coastal areas. This classification shows that the severity of public health security issues between countries with relatively close geographical distances is correlated, and it preliminarily confirms that there is a spatial spillover effect on public health security issues.

**Figure 3:**
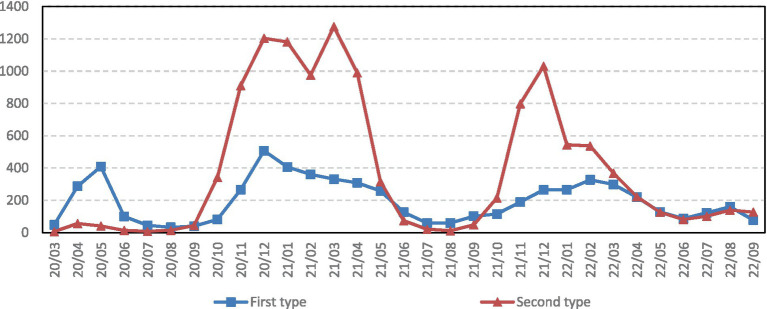
Twenty one months PHS line chart for two types of countries.

According to the clustering results, this study draws a line chart of the PHS of the COVID-19 pandemic in these two types of countries (Figure 3). The changes in PHS during the COVID-19 pandemic vary in different countries, but most of its peaks are concentrated from November 2020 to April 2021. Although the cold weather can inhibit the activity of the virus to a certain extent, the outbreak in winter has brought huge disasters to the residents of all countries due to the unfavorable supervision of the pandemic in European countries. The aggravation of the pandemic situation in European countries in winter also shows that the pandemic is no longer a disaster for one country but a disaster for the whole world. The impact of the spillover effect of the pandemic on neighboring countries is inestimable, and the pandemic should be jointly managed and controlled to prevent its spread. The first category of countries increased significantly in the early stage of the pandemic. It is very likely that this category includes mostly Western European countries with developed tourism, which led to the outbreak of the pandemic due to the flow of tourists in the early stage. After the inflection point in May 2020, it surged after November 2020, but the severity was weaker than that of the second category of countries. It is speculated that the first category of countries had already dealt with the outbreak in the early stage of the pandemic, and so the pandemic control during the second outbreak was more in place than the second category of countries. The second category of countries had relatively mild pandemics before September 2020 and concentrated outbreaks from October 2020 to May 2021, with a sharp increase after October 2021. These countries paid more attention to pandemic supervision in the early stage of the pandemic, such that there was no large-scale outbreak of the pandemic. However, the pandemic was repeated. Owing to the mitigation of pandemic control by the first type of countries, the pandemic broke out in October 2020. At the same time, the countries quickly implemented pandemic supervision and prevention policies such that the COVID-19 pandemic severity gradually decreases after peaking in March 2021. In November 2021, the COVID-19 pandemic severity increased sharply, indicating that the pandemic is threatening and recurrent, and therefore countries should control it scientifically and rationally. After the lockdown was lifted in February 2022, individual countries stopped implementing mandatory closure measures and replaced them with home quarantine measures for sick people. Preventive and control measures are still in place, but they are much more relaxed than they were during the initial period of the pandemic. The economy is also gradually recovering, and the pandemic is steadily declining in both groups of countries. Scientific prevention and control measures can more effectively control the pandemic and promote economic recovery.

**Figure 4:**
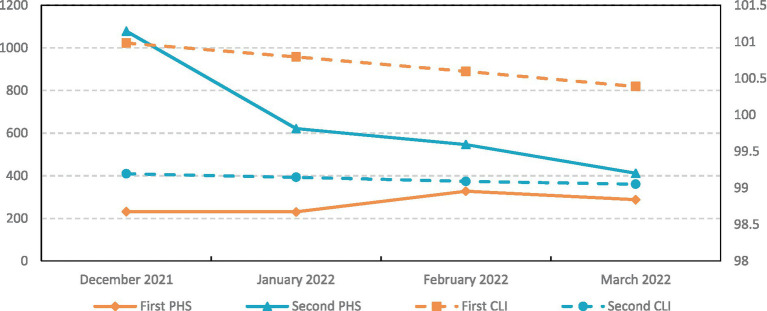
The line chart of changes in PHS and CLI before and after the lift lockdown of the two types of countries.

Since the EU proposed to completely lift the lockdown in February 2022 and proposed new regulations on February 1, tourists only need to carry proof of vaccination, or proof of recovery or proof of negative test to travel unimpeded among the 27 OECD EU countries without isolation or additional coronavirus testing. Therefore, this study selects PHS and CLI before and after the lifting of the lockdown to conduct a preliminary analysis of the rationality of its policy. It selects the PHS of 19 countries from December 2021 to March 2022 for K-means clustering, as shown in Figure 4. The countries are clustered into two categories, and the CLI of these two categories of countries are calculated. From this, it can be concluded that the first type of countries (14 countries, including Austria, Berlin, and Denmark) originally had a relatively low burden of disease but were negatively affected by the lift lockdown policy, with public health security issues minor increase. The second type of countries (5 countries, including the Czech Republic and Sweden) has a relatively serious disease burden, and so they have not been negatively affected by the lift lockdown policy. Instead, due to the open-lift lockdown policy, the domestic pandemic has spilled over to neighboring countries. Governments should continue to be concerned about the spillover effects of public health security issues. On the other hand, the lift lockdown policy has not promoted economic recovery, but rather the CLI has declined in these two types of countries. The economic growth of the first type of countries with a lighter disease burden is better than that of the second type of countries with a heavy disease burden. It shows from the side that lifting the lockdown has not immediately led to economic recovery, and the expected ability to lead to economic recovery has some lag. Improving medical care and reducing national disease burdens can, to some extent, promote economic growth.

### 3.3. Correlation test

In order to measure the correlation between variables, this paper conducted a correlation analysis on explanatory variables and explained variables.

**Figure 5:**
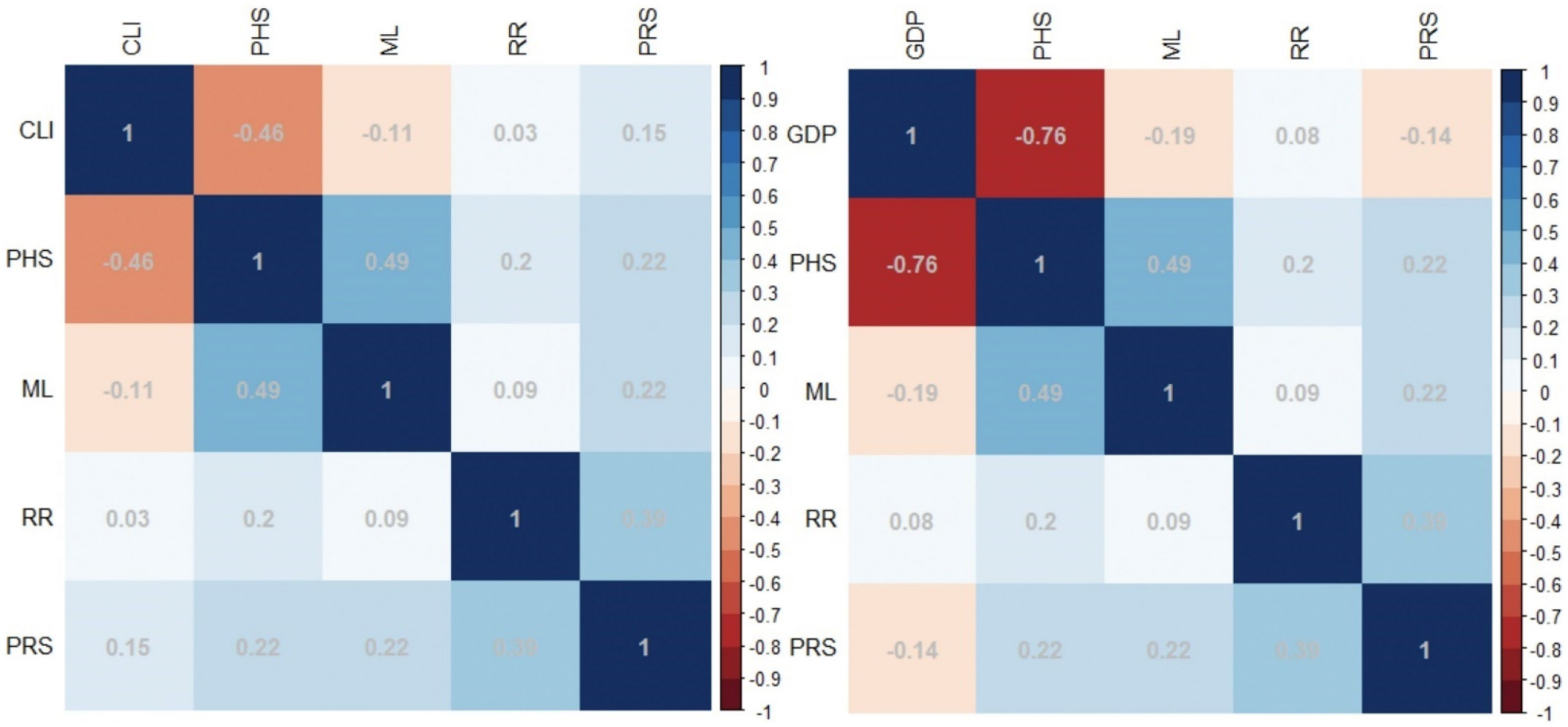
Correlation coefficient chart.

In Figure 5, the variables of public health security and the degree of economic prosperity are negatively correlated, indicating that public health security issues significantly inhibit economic development. The specific relationship between the variables requires further judgment.

### 3.4. Analysis of spatial linkage mechanism

To obtain more robust results, this study adds the time lag term and spatial effect of the degree of economic prosperity to the panel model and uses the dynamic Durbin model to analyze the problems described in the article. To judge which fixed effects the dynamic Durbin model adopts, the study establishes models, respectively. When testing the variable CLI, the log-likelihood value is the maximum when selecting the individual effect model, so it chooses the dynamic Durbin model of the individual effect. When testing the variable GDP, the log-likelihood value is the maximum when selecting the individual effect model, so it chooses the dynamic Durbin model of the individual effect (Table 5).

**Table 5:** Test results of log-likelihood of the fixed-effect dynamic spatial Durbin model.

Variable name	Individual effect	Time effect	Two-way fixed effects
CLI	−323.5015	−860.8148	−992.2393
GDP	616.7979	−997.6296	−830.0706

The time lag item $Y_{t-1}$ of the economic climate in Table 6 is very significant, proving that the above economic climate has the characteristics of time path dependence. The dynamic spatial Durbin model, which takes into account the endogeneity problem and the time lag effect of the degree of economic prosperity, is more reasonable and reflects economic and social development. Public health security issues have no significant impact on the future economic growth of the country. Although the coefficient of the multiplication term of ML and PHS shown in Table 6 is greater than the coefficient of PHS, it is not significant. In terms of spatial spillover effects, public health security issues will have a significant negative effect on the future economic growth of neighboring countries, indicating that a country’s public health security issues will have a negative impact on the future economic growth of neighboring countries. The coefficient of the multiplication term of ML and PHS is significantly positive in terms of spatial spillover, indicating that the improvement of medical level can have a certain mitigation effect on the negative impact of public health security issues in neighboring countries and regions on the degree of future economic prosperity. The coefficient of RR is significantly negative, but there is a significant positive spillover effect. It shows that the increase in RR of the COVID-19 pandemic will aggravate the pandemic and reduce people’s confidence in the future economic climate. Therefore, it will inhibit the future economic climate, but will not be negatively affected by the virus RR of neighboring countries. Similarly, the reason why the PRS coefficient is positive but not significant is most likely that the pandemic is serious and the state has responded positively to it. At the same time, the impact of COVID-19 on residents and the strictness of policy response offset each other, so PRS is not significant.

**Table 6:** The impact of PHS on CLI − the moderating effect of ML.

CLI	Main effect	Spillover effect
PHS	−0.041	−0.107^**^
PHS × ML	0.021	0.244^***^
$Y_{t-1}$	0.664^***^	0.664^***^
RR	−0.121^***^	0.075^**^
PRS	0.054	0.042

**5% significance level, ***1% significance level.

In Table 7, the impact of PHS on GDP is significantly negative and the spatial spillover effect is negative, indicating that increasingly serious public health security issues will inhibit the development of the degree of economic prosperity and have a negative impact on the economic growth of neighboring countries. Similarly, the coefficient of the multiplicative term of ML and PHS has increased and the spillover effect is significantly positive, indicating that the improvement of the medical level can bring a certain mitigation effect to the negative impact of domestic public health security issues on economic climate. Additionally, it can significantly alleviate the negative impact of public health security problems in neighboring countries on economic prosperity. The coefficient of RR is significantly negative, but there is a significant positive spillover effect, indicating that the increase in RR of the COVID-19 pandemic will inhibit economic growth at present but will not necessarily have a negative impact on the economic growth of neighboring countries. Similarly, the coefficient of PRS is positive and has a significant negative spillover effect. It may be that the current response of the government to the pandemic will have a positive impact on economic growth but not on the economic development of neighboring countries.

**Table 7:** The impact of PHS on GDP − the moderating effect of ML.

GDP	Main effect	Spillover effect
PHS	−0.007^*^	−0.012
PHS × ML	0.001	0.048^***^
$Y_{t-1}$	0.443^***^	0.443^***^
RR	−0.018^***^	0.019^***^
PRS	0.021^***^	−0.038^***^

*10% significance level, ***1% significance level.

Combining the results in Tables 6, 7, the multiplicative coefficients of ML and PHS are significantly positive in both models in terms of spatial spillover, indicating that the improvement of medical level can bring a certain mitigation effect to the negative impact of domestic public health security issues on economic climate. Hypothesis 1 is confirmed.

### 3.5. Robustness test

Although the COVID-19 DALY used above can accurately measure the severity of regional public health security problems, the increase in the death rate of COVID-19 is likely to increase people’s panic and accelerate the spread of public health security problems. Therefore, a new column of new variables, the COVID-19 death rate (PHS-death), is added below, which is the number of COVID-19 deaths per 100,000 people. Given this, to test the robustness of the dynamic spatial Durbin model, this research replaces the core explanatory variable (PHS) with the COVID-19 mortality rate (PHS-death) for retesting.

From the perspective of coefficient changes, the medical level can also alleviate the negative impact of the death rate of the COVID-19 pandemic on the future and current economic climates. A spillover effect is observed. Within a country, the increase in mortality does not significantly affect people’s prospects for future economic development, but it will have a significant negative impact on the current economic climate and negatively affect the degree of future economic prosperity of neighboring countries (Table 8). Similar to the above results and weaker than the above, the findings further show that the selection of the DALY of the COVID-19 pandemic as a PHS indicator is more appropriate than the COVID-19 pandemic mortality rate. It also shows that the results of the dynamic spatial Durbin model constructed above are credible and robust.

**Table 8:** Influence of PHS on CLI and GDP–robustness test of the moderating effect of ML.

Dependent variable	Independent variable	Main effect	Spillover effect
CLI	PHS-death	−0.042	−0.108^**^
	PHS-death × ML	0.021	−0.246^***^
	$Y_{t-1}$	0.663^***^	0.663^***^
	RR	−0.121^***^	0.075^**^
	PRS	0.054	0.042
GDP	PHS-death	−0.007^**^	−0.012
	PHS-death × ML	−0.001	0.048^***^
	$Y_{t-1}$	0.442^***^	0.442^***^
	RR	−0.018^***^	0.018^***^
	PRS	0.021^***^	−0.037^***^

**5% significance level, ***1% significance level.

### 3.6. Analysis of the impact mechanism of economic growth on the COVID-19 pandemic

The GDP of coefficient is significantly positive (Table 9), indicating that the faster the economic development, the more serious the public health security issues become. Economic growth cannot alleviate public health security issues. The previous section illustrated that public health security issues can impede economic growth. The two results together confirm Hypothesis 2. The current economy develops faster the virus RR becomes faster. Economic development requires exchange contact, which will become a breeding ground for virus reproduction. But the CLI of the coefficient is significantly negative, indicating that the higher the future economic prosperity degree, the lower the virus RR will be. Due to reasonable and scientific government control measures, people no longer panic about the impending pandemic and are full of confidence in the anticipated economic climate. Therefore, the future economic climate will be improved on this basis, which will inhibit the RR of COVID-19, so that the pandemic can be under control.

**Table 9:** Fixed effect regression model of the impact of economic climate on the COVID-19.

Variable name	Coefficient
CLI	−0.206^***^
GDP	0.600^*^
PHS	−0.145^***^
PRS	0.042

*10% significance level, ***1% significance level.

## 4. Discussion

We have deeply realized the impact of COVID-19 on human daily life. In the context of economic globalization, public health security is not only a problem for individual countries but also a problem for the whole world. Therefore, besides the negative impact on the economy, is there any spillover effect of the COVID-19 pandemic? To better analyze the spillover effects of medical level and public health security issues on the economic climate, this study selects EU countries with close economic ties and geographical distance as the research object. This paper establishes a dynamic Durbin model under the theoretical framework of a moderating effect and then studies the impact of public health security on the current and future economic climate of the region and the moderating effect of the medical level. Furthermore, the research perspective is expanded to the spatial dimension to study the spatial spillover effect. The medical level can adjust the negative impact of COVID-19 on the economy and alleviate the negative impact of public health security issues on the economy of neighboring countries. The degree of economic prosperity will affect public health security issues to a certain extent.

The global economy has been significantly affected by the COVID-19 pandemic. Some studies show that COVID-19 has spillover effects on the global economy. First, the spread of the virus has limited people’s social distance and closed economic activity venues. The national economy is developing slowly. Second, the virus spreads exponentially, which causes consumers and investors to lack confidence in future economic development and makes it difficult for the economy to recover steadily (58). It is consistent with the conclusion of the present study. Public health security issues affect not only the current economic situation but also the future economic development trend.

Our study found that the effect of the medical level on the pandemic and the economy is highly significant. In contrast, the impact of pandemic prevention policies on the pandemic and the economy is not fully significant. Pandemic prevention policies can only lead to economic recovery by indirectly influencing people’s confidence in the future economic climate. Thus, the negative impact of a strict pandemic prevention policy on the economy during the latter part of a pandemic is greater than its positive impact on pandemic prevention and control. In the event of COVID-19, because the impact of the COVID-19 pandemic on European countries is asymmetric, the challenges brought by this asymmetry greatly reduce the effect of joint measures taken at the EU level. Therefore, many scholars proposed that the EU should formulate flexible plans to combat the pandemic (59). For example, EU member states reached a 540 billion Euro rescue measure in early April 2020 and approved a 1.85 trillion Euro budget stimulus plan on December 10, 2020 (60). On April 15, 2020, the president of the European Council and the president of the European Commission jointly proposed the “EU road map” to gradually eliminate restrictive measures in pandemic prevention and control, flexibly control and gradually restore normality to residents’ lives, and restore strict control measures when the infection rate of COVID-19 surges (61). Most scholars agree on the need for a greater policy focus on economic recovery, which is consistent with our findings. Our study confirms that policy response strictness has a positive but insignificant effect on pandemic control. At a time when viruses are weaker, economic recovery is even more important. At present, all countries have an open attitude toward COVID-19, and this practically confirms that strict prevention and control policies are no longer appropriate for implementation.

Our study observes a relationship between the COVID-19 pandemic and the degree of economic prosperity. The aggravation of the pandemic will slow down economic growth. If we want to improve the economic climate, we must first improve people’s confidence in the degree of future economic prosperity, to focus on economic recovery in the post-pandemic era. Taking COVID-19 as an example, we must take a compromise between economic growth and public health security issues control. It will improve the future economic climate, control current public health security issues with a long-term perspective, and achieve long-term economic growth.

## 5. Conclusion

Our research shows that public health security issues in one country have spillover effects on the economic development of neighboring countries. Severe public health security issues can hurt the economic development of neighboring countries. At the same time, the development of the medical level can not only alleviate the negative effect of the pandemic on the economy in one country but also alleviate the negative effect of the pandemic on the economy in neighboring countries.

### 5.1. Theoretical contributions

At the moment of the pandemic, countries with high medical levels can better cope with the medical burden brought by the pandemic and effectively alleviate the negative effects of the pandemic. After reading the relevant literature, we found a few articles on the impact of the severity of the COVID-19 pandemic in a country on its neighboring countries, but this was very important in the context of economic globalization. The EU is a political and economic community. It has significant outbreak spillover effects. Therefore, this paper chooses the EU, a region with strong economic cooperation, for research. The 19 countries cited in this article are all from the EU. As a political and economic community, the EU promotes the development of countries in the EU through the implementation of treaties and plans. There are close political and economic exchanges between countries, so it is easier to transmit public health security issues. In order to study the spatial linkage problems encountered in adjacent regions when facing public health and safety problems, this paper introduces the moderating effect theory into the dynamic spatial Durbin model to explain the spillover effect of interregional public health security issues and the moderating role of the medical level. The aggravation of the pandemic in one country is very likely to cause pandemic burdens to neighboring countries. Similarly, the high level of national medical care can provide medical assistance to more patients, reducing the scope of public health security problems in the region. At the same time, it provides medical assistance to neighboring countries to alleviate the negative impact on economic growth trends of neighboring countries due to the spread of the public health security issues pandemic. Previous studies have often discussed the direct effect of the pandemic on the economy and other aspects. However, few studies have studied the spillover effect of the pandemic on the economy and the direct and indirect moderating effects of the medical level. In the process of globalization, countries connect more closely, and the spillover effects will become stronger. This research provides a new perspective and method of interregional linkages for future research on public health security.

### 5.2. Recommendations

Our research shows that the impact of a public health security problem is extensive, which will affect surrounding countries due to spillover effects. The improvement of medical level can help the country and even neighboring countries resist the attack of public health security problems. It is clear that the medical level is very important in any public health security issue, and therefore the government needs to maintain the medical system in such issues. For example, enhancing the health protection of healthcare workers. Some countries can provide medical assistance, such as medical supplies, personnel, and programs, to neighboring countries when the pandemic is controllable in their own. Our study finds that policy response strictness has a positive but insignificant effect on public health security and the economy throughout the outbreak period. It is because strict pandemic prevention policies are more effective in controlling the spread of the pandemic when the virus is virulent in the early stages of COVID-19. However, as the virus species mutates, the lethality rate of the pandemic decreases significantly, and strict pandemic prevention policies are less effective in controlling the pandemic but harm socioeconomic recovery. For example, the public health security issues do not intensify after the implementation of the lift lockdown policy in the EU. The intensity of pandemic control needs to base on the severity of the public health security issues and the intensity of pandemic transmission. Therefore, in the face of public health security issues, we recommend strict control in the early stages when the virus is strong. In the later period, control measures should be gradually relaxed. Eventually, economic recovery will be achieved.

In this study, 19 countries in the EU were selected as subjects. When selecting other countries, it is necessary to consider the similarity of their political and economic systems. The presence of large political and economic differences among neighboring countries will most likely lead to weaker spillover effects in neighboring countries than in non-neighboring countries. Therefore, it is necessary to take into account the inter-country differences in the geographic distance and economic level matrix when considering the public health security issues’ spillover effects among countries with large political and economic differences. When discussing the impact of the COVID-19 pandemic on the economy, this study introduced policy response strictness and virus RR as control variables. However, in real life, there are still many variables that can affect the severity of the COVID-19 pandemic and economic growth, such as psychological factors (i.e., people’s fear of the COVID-19 pandemic). These variables are difficult to quantify, so more precise frameworks need to be constructed to measure people’s psychological factors. There is still room for improvement in the selection of control variables in this study.

### 5.3. Future research perspectives

In terms of research methodology, the spatial Durbin model relies on the spatial matrix, and the study of other factors that affect the spatial matrix can make the spatial Durbin model more accurate. At the same time, more control variables should be introduced in future studies to improve the credibility of the model results. In terms of research content, the COVID-19 of the outbreak impact on all types of economies varies in degree. For the tourism, catering, and retail industries, COVID-19 deals a severe blow. The travel companies, hotels, airports, and train stations have taken a hit large due to a drastic decrease in travel demand. The restaurant companies are facing reduced patronage and operational difficulties due to the ongoing pandemic. The brick-and-mortar stores in the retail industry have seen a decrease in patronage and sales due to the ongoing pandemic. For the healthcare and online economies, the pandemic is both a hardship and an opportunity. During the pandemic, sales in these industries grow as people spent most of their time at home, leading to an increase in demand for home entertainment and digital products. Many gaming industries experience significant economic benefits from the pandemic situation. In addition, the healthcare industry also sees growth due to the ongoing pandemic. Therefore, in future research, we can analyze the economic development of different industries under the background of the COVID-19 pandemic. COVID-19 has significantly impacted the global unemployment rate. Once a country’s unemployment rate becomes too high, the society is likely to become unstable. To cope with the effects of COVID-19, the government must take measures, and the purpose of our study is to provide suggestions to address the negative impacts of the COVID-19 crisis. It can even extend the pandemic to global public health and security events, enabling scholars to find the commonalities of economic development under various public health and security events. General conclusions can be drawn in the face of global changes, and future public health and security events must be prepared in advance.

## Data availability statement

Publicly available datasets were analyzed in this study. This data can be found here: https://github.com/owid/covid-19-data/tree/master/public/data.

## Ethics statement

Ethical review and approval was not required for the study on human participants in accordance with the local legislation and institutional requirements. Written informed consent for participation was not required for this study in accordance with the national legislation and the institutional requirements.

## Author contributions

RF made substantial contributions and participated in all aspects of the paper, conducted the methodology, analyzed the data, and wrote the manuscript. All authors listed have made a substantial, direct, and intellectual contribution to the work and approved it for publication.

## Funding

This research was funded by the National Social Science Fund of China, grant number 20BTJ005.

## Conflict of interest

The authors declare that the research was conducted in the absence of any commercial or financial relationships that could be construed as a potential conflict of interest.

## Publisher’s note

All claims expressed in this article are solely those of the authors and do not necessarily represent those of their affiliated organizations, or those of the publisher, the editors and the reviewers. Any product that may be evaluated in this article, or claim that may be made by its manufacturer, is not guaranteed or endorsed by the publisher.
